# Isolation of Live Premature Senescent Cells Using FUCCI Technology

**DOI:** 10.1038/srep30705

**Published:** 2016-08-09

**Authors:** Danli Wang, Ping Lu, Yang Liu, Li Chen, Rui Zhang, Weihao Sui, Alexandru George Dumitru, Xiaowen Chen, Feiqiu Wen, Hong-Wei Ouyang, Junfeng Ji

**Affiliations:** 1 Dr. Li Dak Sum & Yip Yio Chin Center for Stem Cell and Regenerative Medicine, School of Medicine, Zhejiang University, Hangzhou, Zhejiang Province, China; 2 Department of Pathophysiology, Anhui Medical University, Hefei 230032, China; 3 Division of Hematology and Oncology, Shenzhen Children’s Hospital, 7019 Yitian Road, Futian District, Shenzhen, China; 4 Zhejiang Provincial Key Laboratory of Tissue Engineering and Regenerative Medicine, Hangzhou, China

## Abstract

Cellular senescence plays an important role in diverse biological processes such as tumorigenesis and organismal aging. However, lack of methods to specifically identify and isolate live senescent cells hampers the precise understanding of the molecular mechanisms regulating cellular senescence. Here, we report that utilization of fluorescent ubiquitination-based cell cycle indicator (FUCCI) technology allows isolation of live premature senescent cells induced by doxorubicin treatment. Exposure of human foreskin fibroblasts (HFFs) to a low dose of doxorubicin led to cellular senescent phenotypes including formation of γ-H2AX and 53BP1 foci indicative of DNA damage, decreased cell proliferation and increased senescence-associated β-galactosidase (SA-β-gal) activity. Importantly, doxorubicin-induced senescent cells were arrested at S/G2/M phases of cell cycle which can be reported by a construct encoding a fragment of hGeminin fused with monomeric Azami-Green (mAG-hGeminin). Flow cytometric sorting of GFP^+^ cells from doxorubicin-treated HFFs carrying mAG-hGeminin reporter enabled isolation and enrichment of live senescent cells in the culture. Our study develops a novel method to identify and isolate live premature senescent cells, thereby providing a new tool to study cellular senescence.

Cellular senescence, originally described as “the Hayflick Limit” of human diploid fibroblasts during replication *in vitro*, denotes an irreversible cell cycle arrest and stable loss of proliferative capacity of mammalian cells^1^. Apart from replicative senescence caused by telomere shortening^2^,^3^, cellular senescence is now found to be triggered by various forms of stress such as oncogene activation^4^, DNA replication stress^5^, DNA-damaging chemotherapeutic reagents^6^, which are collectively termed premature cellular senescence. Despite different categories of cellular senescence, senescent cells are currently defined by a common set of features including cell cycle arrest, morphological changes and increased level of lysosomal β-galactosidase (SA-β-gal) activity^7^. SA-β-gal staining has been widely used to identify senescent cells and C_12_FDG, a fluorogenic substrate for SA-β-gal activity, has been developed to identify and sort live senescent cells by flow cytometry^8^,^9^. However, certain non-senescent cells also exhibit increased SA-β-gal activity^10^, suggestive of non-specificity drawback of the SA-β-gal staining method. Activation of tumor suppression networks comprising p53, p21 and p16^INK4A^-RB signal transduction pathway has been well documented in senescent cells^7^. Interestingly, lamin B1 loss has been recently reported to serve as a biomarker of senescent cells^11^. However, these methods do not allow identification and isolation of live senescent cells. Thus, simple methods to specifically identify and, in particular, isolate senescent cells are still lacking, which impedes precise understanding of the biological functions of cellular senescence despite its well-known role in tumorigenesis, organismal aging and embryogenesis^12^,^13^,^14^. In this study, we employed fluorescent ubiquitination-based cell cycle indicator (FUCCI) system to identify and isolate premature senescent cells induced by doxorubicin treatment *in vitro* and developed a novel tool to study cellular senescence.

## Results

### Doxorubicin treatment induced senescence of HFFs

**Figure 1:**
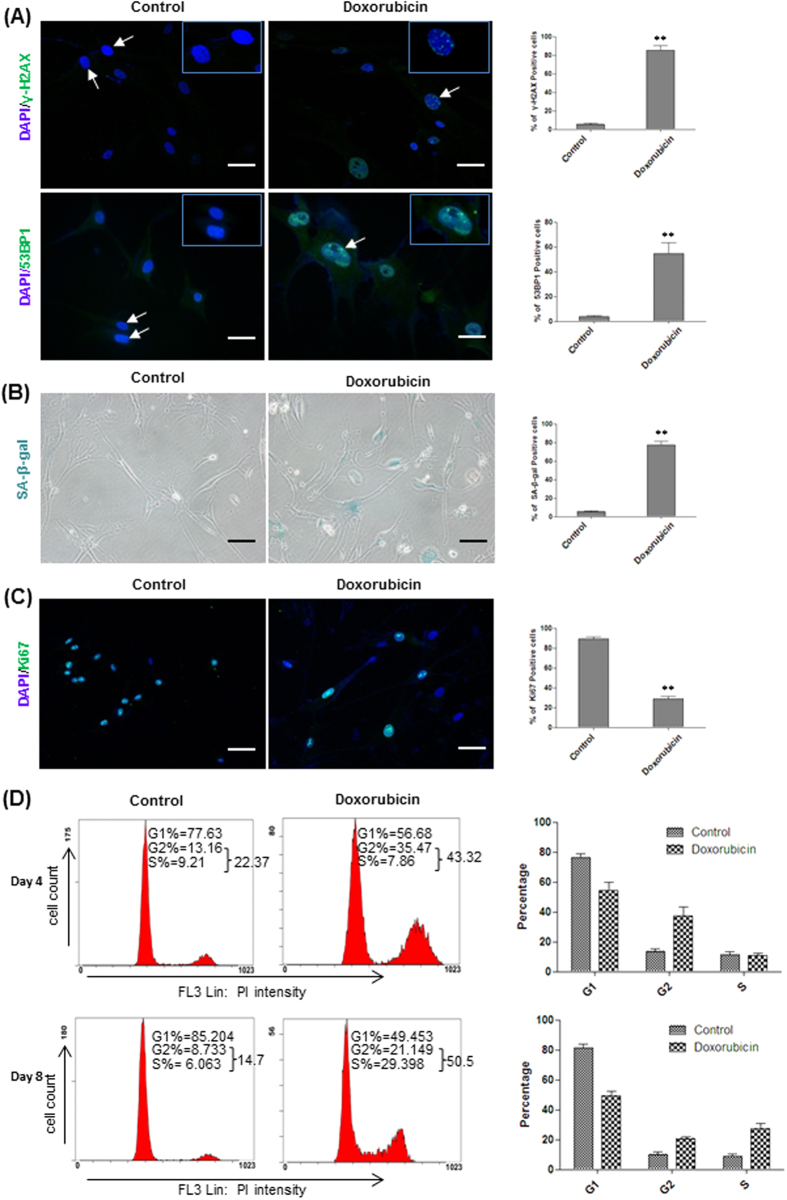
Doxorubicin treatment induced premature senescence of HFFs. (**A**) Left, control HFFs and HFFs treated with doxorubicin for 4 days were stained with by γ-H2AX and 53BP1 antibodies, respectively. Right, the percentages of γ-H2AX and 53BP1 positive cells were quantified (**p < 0.01, t-test, n = 3). Scale bar: 50 μm. Cells in the inserts of the images were shown by arrows. (**B**) Left, SA-β-gal staining was performed on control HFFs and HFFs after 4 days of doxorubicin treatment. Right, the percentages of SA-β-gal positive cells were quantified (**P < 0.01, t-test, n = 3). Scale bar: 200 μm. (**C**) Left, Ki67 staining was performed on control HFFs and HFFs after 4 days of doxorubicin treatment. Right, the percentages of Ki67 positive cells were quantified (**p < 0.01, t-test, n = 3). Scale bar: 100 μm. (**D**) Control HFFs and HFFs after 4 and 8 days of doxorubicin treatment were performed with flow cytometry analysis. The fractions of cells at G1, S and G2 phases were quantified (n = 3).

We used doxorubicin, a widely used chemotherapeutic reagent, to induce senescence of human fibroblasts. We first tested the effect of doxorubicin treatment on the growth of human foreskin fibroblasts (HFFs). Exposure of HFFs to doxorubicin reduced the cell number in a dose-dependent manner (Fig. S1A), suggesting that doxorubicin treatment caused cell death and/or cellular senescence as previously reported^15^,^16^. Treatment of HFFs with doxorubicin at the concentration of 100 ng/ml for 12 hours robustly inhibited the cell growth without causing obvious cell death and we used this concentration for the rest of this study (Fig. S1A). Immunostaining results showed that, in contrast to the control, doxorubicin treatment induced the formation of 53BP1 and γ-H2AX foci in the nucleus indicative of DNA damage (Fig. 1A). The DNA damaging effect of doxorubicin is likely due to that doxorubicin intercalates DNA and suppresses the progression of topoisomerase II thereby preventing DNA replication^17^. In line with this, doxorubicin treatment significantly inhibited proliferation and caused cellular senescence as shown by Ki67 and SA-β-gal staining, respectively (Fig. 1B,C). Co-staining of Ki67 and P21 showed that there were significantly higher number of Ki67^−^P21^+^ cells in doxorubicin-treated HFFs than that in the control (Fig. S1). Our results are consistent with previous evidence reporting that Ki67, a widely used cell proliferation marker, decreases in senescent cells^18^,^19^,^20^. Another hallmark of cellular senescence is morphological change which is likely driven by cytoskeleton remodeling^21^,^22^. To look into this, we performed immunostaining of β-tubulin, phallodin and vimentin at 4 and 8 days of doxorubicin treatment. Our results showed that doxorubicin treatment caused cytoskeleton remodeling which may contribute to the morphological changes such as irregular and larger size of the nuclei and bigger and flattened cell size reminiscent of senescent phenotype (Fig. 1C and Fig. S2B). Furthermore, DNA content analysis by flow cytometry demonstrated that HFFs treated with doxorubicin were irreversibly blocked at S/G2/M phases of the cell cycle 4 and 8 days after treatment, respectively (Fig. 1D). Taken together, our results showed that doxorubicin treatment caused DNA damage which led to premature senescent phenotypes of HFFs arrested at S/G2/M phases of the cell cycle.

### Isolation of live premature senescent HFFs carrying mAG-hGeminin reporter

**Figure 2:**
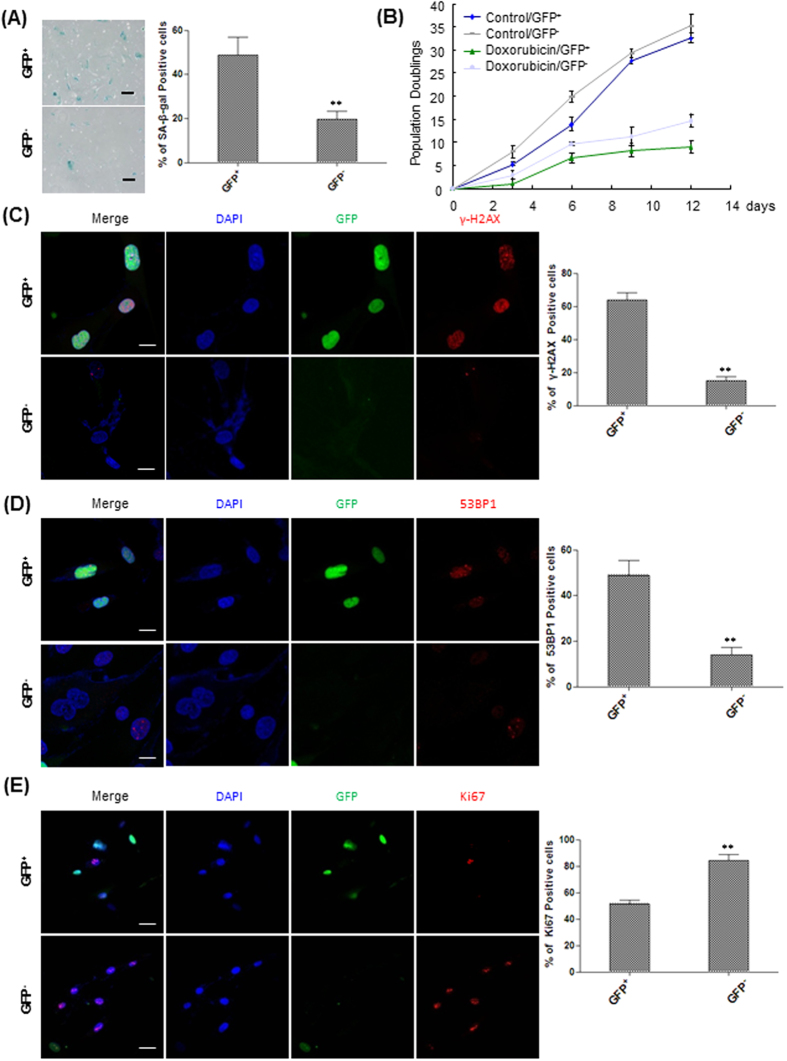
Isolation of live premature senescent HFFs carrying mAG-hGeminin reporter. (**A**) Left, SA-β-gal staining of GFP^+^ and GFP^−^ cells sorted from HFFs carrying mAG-hGeminin reporter at day 8 of doxorubicin treatment. Right, the percentages of SA-β-gal positive cells were quantified (**P < 0.01, t-test, n = 3). Scale bar: 200 μm. (**B**) Growth curve analysis of GFP^+^ and GFP^−^ cells sorted from HFF cells carrying mAG-hGeminin reporter at day 8 of treatment with or without 100 ng/ml doxorubicin. Cells were seeded into 12-well plates and counted at the indicated days. Long-term cumulative population doubling was determined by passaging cells every 3 days. Population doublings were calculated at different passages and plotted. The p values between Control/GFP^+^ and Doxorubicin/GFP^+^ were <0.01 at all days examined except at day 3 (*p* = 0.0475, t-test, n = 3). The p values between Control/GFP^−^ and Doxorubicin/GFP^−^ were all <0.01 except on day 3 (*p* = 0.07, t-test, n = 3). (**C**) Left, GFP^+^ and GFP^−^ cells sorted from HFFs carrying mAG-hGeminin reporter at day 8 of doxorubicin treatment were stained with by γ-H2AX antibody. Right, the percentages of γ-H2AX positive cells were quantified (**p < 0.01, t-test, n = 3). Scale bar: 30 μm. (**D**) Left, GFP^−^ and GFP^−^ cells sorted from HFFs carrying mAG-hGeminin reporter at day 8 of doxorubicin treatment were stained with by 53BP1 antibody. Right, the percentages of 53BP1 positive cells were quantified (**p < 0.01, t-test, n = 3). Scale bar: 30 μm. (**E**) Left, GFP^+^ and GFP^−^ cells sorted from HFFs carrying mAG-hGeminin reporter at day 8 of doxorubicin treatment were stained with by Ki67 antibody. Right, the percentages of Ki67 positive cells were quantified (**p < 0.01, t-test, n = 3). Scale bar: 50 μm.

To isolate doxorubicin-induced senescent cells arrested at S/G2/M phases of the cell cycle, we utilized fluorescent ubiquitination-based cell cycle indicator (FUCCI) system. Functioning as a set of fluorescent probes which enable the visualization of cell cycle progression in living cells, FUCCI takes advantage of the highly selective, rapid degradation of the replication licensing factors mediated by the ubiquitin-proteasome system^23^. A fusion protein of a fragment of Cdt1 with the fluorescent protein monomeric Kusabira-Orange 2 (mKO2) serves as an indicator of G1 phase, whereas a fragment of Geminin fused with the fluorescent protein monomeric Azami-Green (mAG) allows visualization of cells at S, G2 and M phases^23^. Therefore, we used mAG-hGeminin indicator to visualize doxorubicin-induced premature senescent cells which were blocked at S/G2/M phases. Transduction of normal HFFs with viruses carrying mAG-hGeminin reporter allowed visualization of GFP^+^ cells under microscopy and further flow cytometry analysis confirmed that GFP^+^ cells predominantly resided in S/G2/M phases of the cell cycle (Fig. S3A). Doxorubicin treatment of HFFs carrying mAG-hGeminin reporter also robustly inhibited cell growth (Fig. S3B) and cellular senescence as shown by SA-β-gal staining (Fig. S3C). Similarly, flow cytometry analysis further demonstrated that doxorubicin treatment of HFFs carrying mAG-hGeminin reporter significantly increased the percentage of cells in S/G2/M phases with a corresponding increase in the fraction of GFP^+^ cells after 4 and 8 days of treatment, respectively, which indicated cell cycle arrest at S/G2/M phases (Fig. S3D). We then performed flow cytometric sorting of senescent cells. In order to minimize isolation of non-senescent cells, we sorted out GFP^+^ HFFs at day 8 of doxorubicin treatment as a very low fraction of control cells without doxorubicin treatment were in S/G2/M phase due to contact inhibition when the cells reached confluence at that stage. After sorting, SA-β-gal staining confirmed that significant more senescent cells were found in the culture established from the isolated GFP^+^ cells in comparison with the GFP^−^ cells (Fig. 2A). To evaluate the long-term cell proliferation capacity, we performed population doubling analysis on the GFP^+^ and GFP^−^ cells isolated from both control and doxorubicin-treated groups. No significant difference in cell growth was observed between the GFP^+^ and GFP^−^ cells isolated from the untreated control cells (Fig. 2B), ruling out the potential effect of mAG-hGeminin on cell proliferation. In contrast, there was significant less proliferation of the GFP^+^ cells isolated from doxorubicin-treat group than that sorted from the control at all days examined (Fig. 2B). γ-H2AX and 53BP1 immunostaining of sorted cells showed that there were significantly more cells possessing γ-H2AX and 53BP1 foci in the GFP^+^ fraction compared to the GFP^−^ subset (Fig. 2C,D). This was consistent with the significantly lower proliferation in the sorted GFP^+^ cells as indicated by Ki67 staining (Fig. 2E). Therefore, taken together, it demonstrated that isolation of GFP^+^ cells from mAG-hGeminin-transduced HFFs after doxorubicin treatment enriched senescent cells in the culture.

## Discussion

Characterized by a modest increase in the number of SA-β-gal positive cells, cellular senescence in proliferating cells shows a permanent arrest of the cell cycle that can be triggered by a variety of stressors including DNA-damaging agents doxorubicin^24^. In this study, we showed that doxorubicin treatment induced senescent phenotypes of HFFs with the cell cycle arrest at S/G2/M phases. Senescent cells stably arrested at S/G2/M phases can be reported by the mAG-hGeminin system, which allowed us to further isolate live senescent cells. Our FACS sorting results showed that GFP^+^ fraction was significantly enriched with more senescent cells than the GFP^−^ fraction, supporting that FUCCI system can be utilized to isolate and enrich live senescent cells *in vitro*. However, our results also revealed the presence of γ-H2AX and 53BP1 foci positive cells in the sorted GFP^−^ fraction and Ki67 positive cells in the GFP^+^ fraction, respectively (Fig. 2C,E). This could be because the viral infection efficiency of HFFs with mAG-hGeminin reporter was unlikely 100% and some senescent cells may not carry this reporter therefore being sorted into GFP^−^ fraction. On the other hand, a fraction of non-senescent cycling cells was present in the doxorubicin-treated culture and they will be likely sorted into the GFP^+^ fraction if they carried mAG-hGeminin reporter and were progressing through S/G2/M phase at the time of sorting. Therefore, it is likely the isolated GFP^+^ cells included a small fraction of non-senescent cycling cells. Despite the technical limitations, there was still significant higher percentage of senescent cells in the sorted GFP^+^ than the GFP^−^ fraction based on the senescence characterization results (Fig. 2). Therefore, it supports that FUCCI technology can be used to isolate and enrich live senescent cells. To our knowledge, our study represents the first report to isolate and enrich live premature senescent cells other than SA-β-gal-based method^9^. Although utilization of this system in other *in vitro* and *in vivo* senescence models remains to be further investigated, we provide a proof-of-concept study to develop a novel method to identify and isolate live senescent cells, thereby providing a useful tool to further understand the molecular mechanisms underlying cellular senescence and the biological roles it plays in future.

## Methods

### Cell culture

Human neonatal foreskin fibroblasts (HFFs) (ATCC) were maintained in DMEM high glucose media (Corning) supplemented with 10% HyClone fetal bovine serum (Thermo Scientific) at 37 °C with 5% CO_2_. Cells were seeded at the density of 1 × 10^3^/cm^2^ in 10 cm culture dish before treatment. 48 h after seeding, cells were incubated in complete medium supplemented with different concentrations of doxorubicin (Sigma-Aldrich) for 12 h. Cells were then cultured in fresh complete medium with regular medium change and subjected to staining and flow cytometry analysis after 4 and 8 days of treatment.

### SA-β-gal staining

Cells were seeded in 6-well plate and then washed twice with phosphate-buffered saline (PBS). Cells were fixed with 0.2% glutaraldehyde for 10 minutes at room temperature, washed twice with PBS, and then stained with X-gal staining solution (1 mg/ml X-gal, 40 mmol/l citric acid/sodium phosphate, 5 mmol/l potassium ferricyanide, 5 mmol/l potassium ferrocyanide, 150 mmol/l NaCl, 2 mmol/l MgCl_2_) at pH 6.0 overnight. Images were captured with the Olympus IX71 microscope (10 × magnification). SA-β-gal positive cells were counted in 3–5 randomly selected images and the percentages of SA-β-gal positive cells were averaged and quantified for statistical analysis.

### Lentivirus production and cell transduction

mAG-hGeminin plasmid was kindly provided by Dr. Atsushi Miyawaki. This lentiviral plasmid was co-transfected together with gag/pol, REV and vesticular stomatitis virus G protein packaging plasmids into 293 T cells. 48 h after transfection, viral supernatants were collected, filtered through 0.45-μm syringe filters and then were used to transduce cells at about 70% confluence in the presence of 10 ug/ml hexadimethrine bromide (Sigma Aldrich).

### Immunecytochemistry

Cells were fixed with 4% formaldehyde for 30 minutes, washed with PBS for 5 min twice, and then blocked with PBS containing 1% BSA and 1% triton for 1 h at room temperature. Cells were then incubated with polyclonal γ-H2AX (Cell Signaling), 53BP1 (a gift from Dr. Jun Huang’s laboratory at Life Science Institute, Zhejiang University), β-tubulin (Cell signaling), phalloidin (Life Technology), vimentin (Dakocytomation), Ki67 (Abcam) and P21 (Cell Signaling) antibodies diluted in PBS containing 1% BSA for 2 h at room temperature. Cells were then washed with PBS and stained with a DyLight 488 goat anti-rabbit secondary antibody at 1/500 dilution. Nuclei were counterstained with DAPI (5 ug/ml; Beyotime, China). Images were captured with a laser scanning confocal microscope (Olympus DP70).

### Cell cycle analysis

About 2 × 10^6^ cells were fixed with cold 70% ethanol for 1 h at 4 °C. Cells were then incubated in freshly prepared staining solution consisting of 0.1% Triton X-100 (Sigma), 5 ug/ml PI, and 50 ug/ml DNAse-free RNAse A (Sigma) for 30 minutes at 37 °C. Cells were then analyzed on flow cytometer. Cell cycle distribution was determined with the MultiCycle AV-DNA Analysis program.

### Population doubling assay

10,000 cells/well were plated in 12-well plate and cells were then passaged every 6 days. At each passage, cells were counted by a hemocytometer. Population doubling levels (PDL) were calculated using the equation PD = log(Nf/N0)/log2 where Nf equals the number of final cells and N0 equals the number of initial cells. Cumulative PDLs were calculated by summing the PDLs from all passages. Data were expressed as cumulative PDL from 3 independent experiments.

### Statistical analyses

The results were presented as mean ± SEM of minimum three independent experiments. Statistical significance was determined by student’s t-test. Differences with *p* < 0.05 were considered significant.

## Additional Information

**How to cite this article**: Wang, D. *et al*. Isolation of Live Premature Senescent Cells Using FUCCI Technology. *Sci. Rep.* **6**, 30705; doi: 10.1038/srep30705 (2016).

## Supplementary Material

Supplementary Information
