# Supplementary material for: Isolation of Live Premature Senescent Cells Using FUCCI Technology
Source: Sci Rep. 2016 Aug 9;6:30705. doi: 10.1038/srep30705 (PMC4977570; doi:10.1038/srep30705)
Supplement: Supplementary Information [file srep30705-s1.pdf]

Isolation of Live Premature Senescent Cells Using FUCCI Technology

Danli Wang[1, 4], Ping Lu[1, 4], Yang Liu[1], Li Chen[1], Rui Zhang[1] , Weihao Sui[1], Alexandru George Dumitru[1] , Xiaowen Chen[3], Feiqiu Wen[3*], Hong-Wei Ouyang[1, 2*], Junfeng Ji[1, 2*]

Supplementary Figure Legends

Figure S1. Co-staining of P21 and Ki67 in HFFs with/without doxorubicin treatment.

(A) Representative fluorescent images showing the co-staining of P21 (green), Ki67(red foci) and DAPI(blue) in control and doxorubicin-treated HFFs at day 4. Arrows showed respective P21 and Ki67 positive cells and inserts showed those cells at higher magnification. Scale bar, 100 μm, n = 3.

(B) Quantification of the control and doxorubicin-treated cells co-stained with P21 and Ki67. **, $P < 0.01$; *, $P < 0.05$; Results represented average ± SEM

Figure S2. Doxorubicin treatment inhibits growth of HFFs in a dose-dependent manner, and causes cytoskeleton remodeling of HFFs at a low dose.

(A) $1 \times 10^3/cm^2$ cells were seeded in 10 cm dish, cultured for 48h, and then treated with different concentrations of doxorubicin for 12h. Cells were counted at the indicated time points, n = 3.

(B) Cells at days 4 and 8 of doxorubicin treatment (100 ng/ml) were stained with by β-tubulin, phalloidin and vimentin. Scale bar, 50μm.

Figure S3. Doxorubincin treatment induces premature senescence of HFFs carrying mAG-hGeminin reporter.

(A) Microscopic and cell cycle analysis of HFFs transduced with lentivirus carrying mAG-hGeminin reporter. Scale bar: 200μm.

(B) The numbers of cells carrying mAG-hGeminin reporter treated with or without 100 ng/ml doxorubicin were counted at days 4 and 8, n = 3.

(C) SA-β-gal staining of HFFs carrying mAG-hGeminin reporter treated with or without 100 ng/ml doxorubicin at day 4. Pictures were taken randomly and senescent cells in each field were independently counted. The percentages of SA-β-gal positive cells were quantified and results represented average ± SEM (** $p<0.01$, t-test, n=3). Scale bar: 200μm.

(D) Cell cycle analysis of mAG-hGeminin-transduced cells at days 4 and 8 of treatment with or without 100 ng/ml doxorubicin (n=3).

Figure. S1

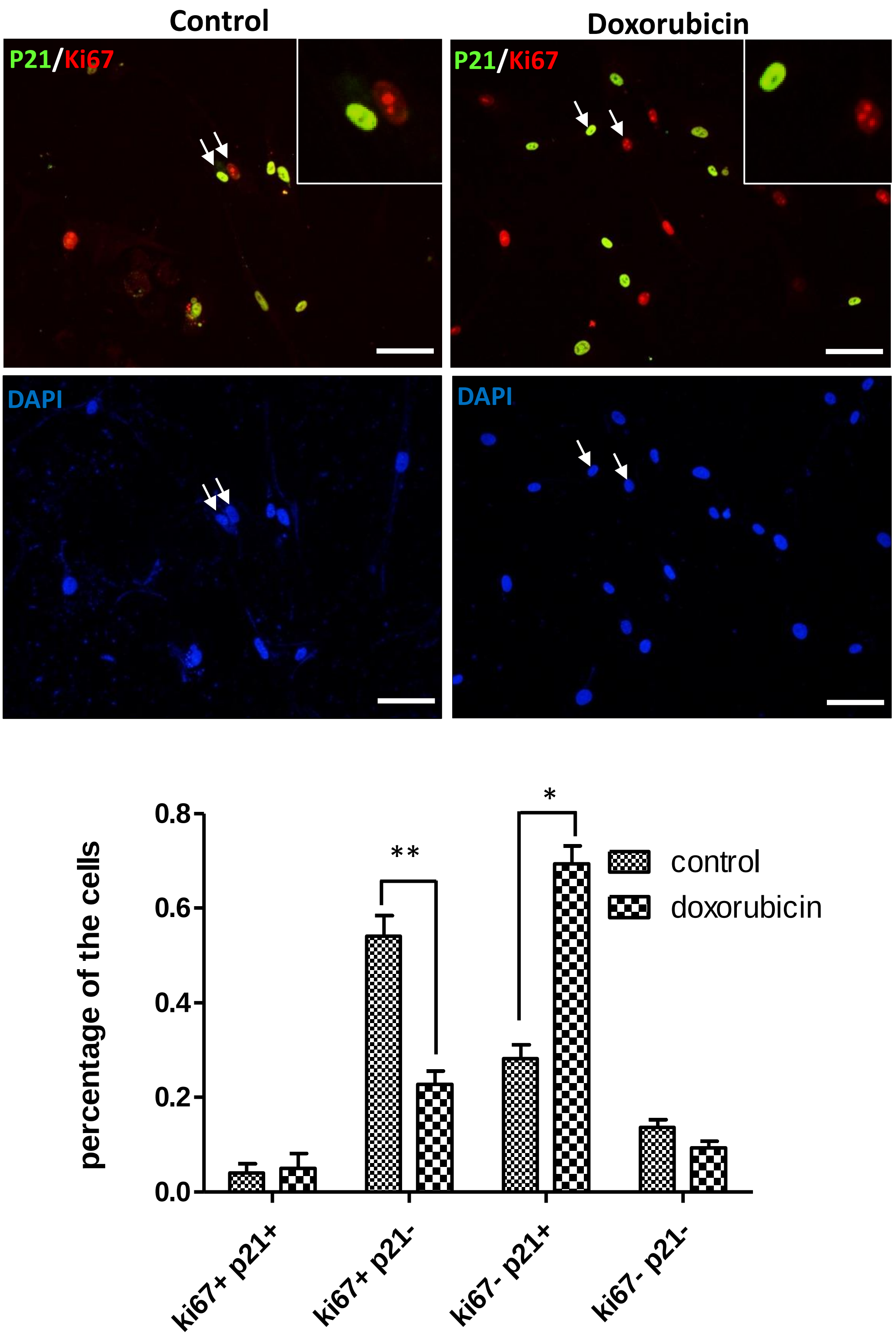

Control
Doxorubicin
P21/Ki67
P21/Ki67
DAPI
DAPI
percentage of the cells
0.8
0.6
0.4
0.2
0.0
**
*
control
doxorubicin
ki67+ p21+
ki67+ p21-
ki67- p21+
ki67- p21-

Figure. S2

(A)

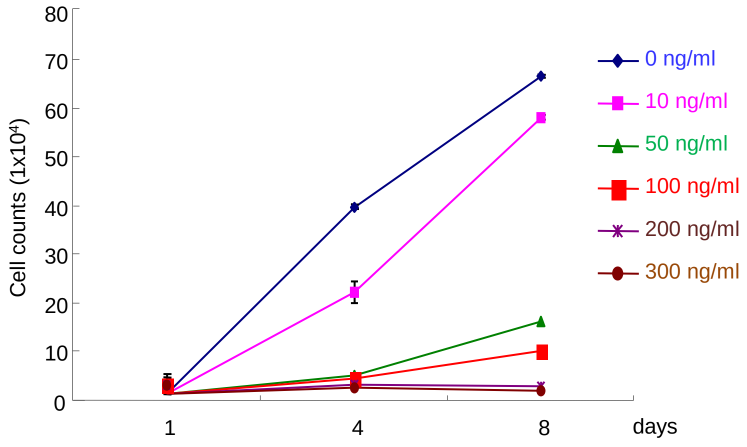

Cell counts (1x10⁴)
80
70
60
50
40
30
20
10
0
1
4
8
days
0 ng/ml
10 ng/ml
50 ng/ml
100 ng/ml
200 ng/ml
300 ng/ml

(B)

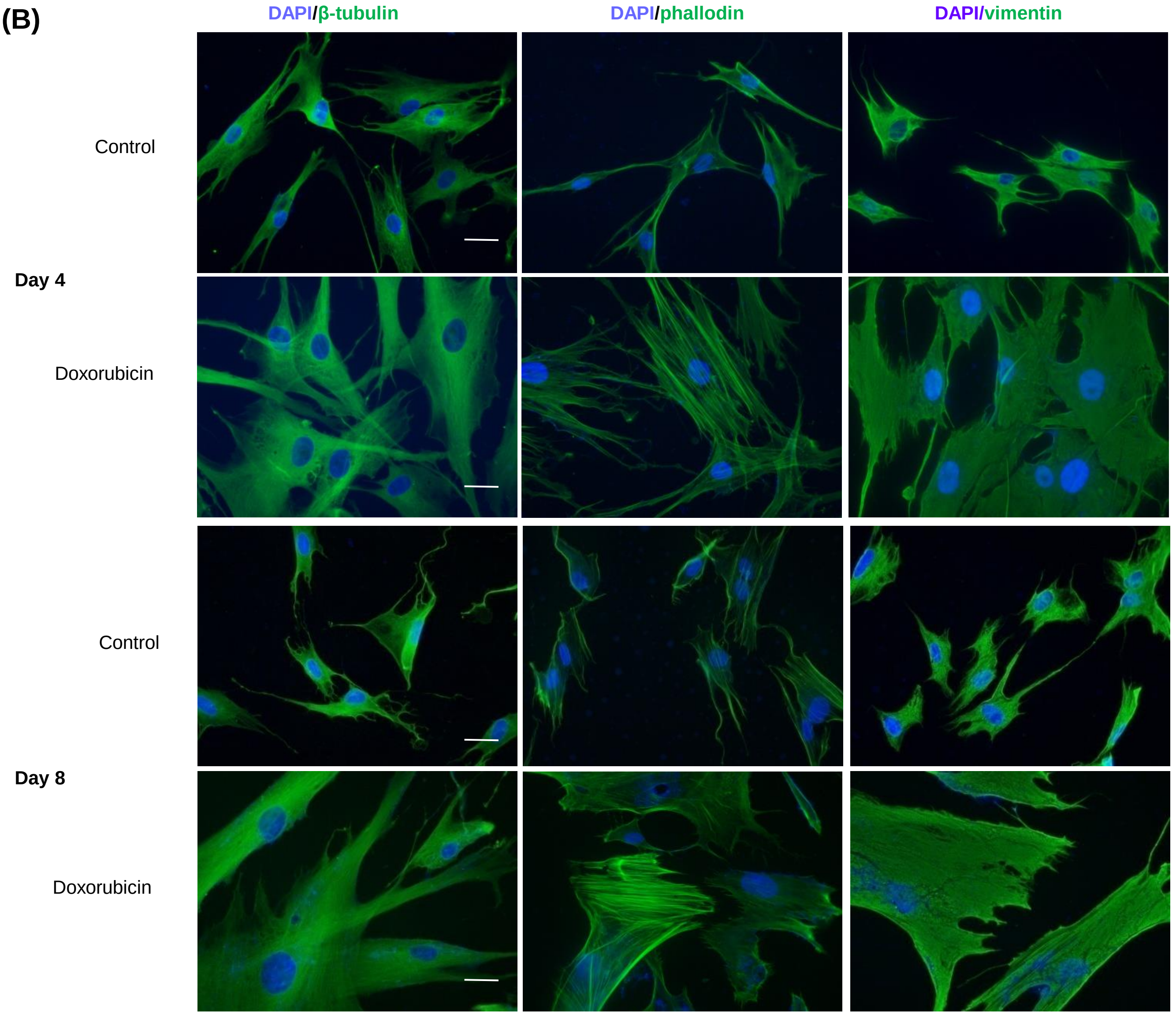

DAPI/β-tubulin
DAPI/phallodin
DAPI/vimentin
Control
Day 4
Doxorubicin
Control
Day 8
Doxorubicin

Figure. S3

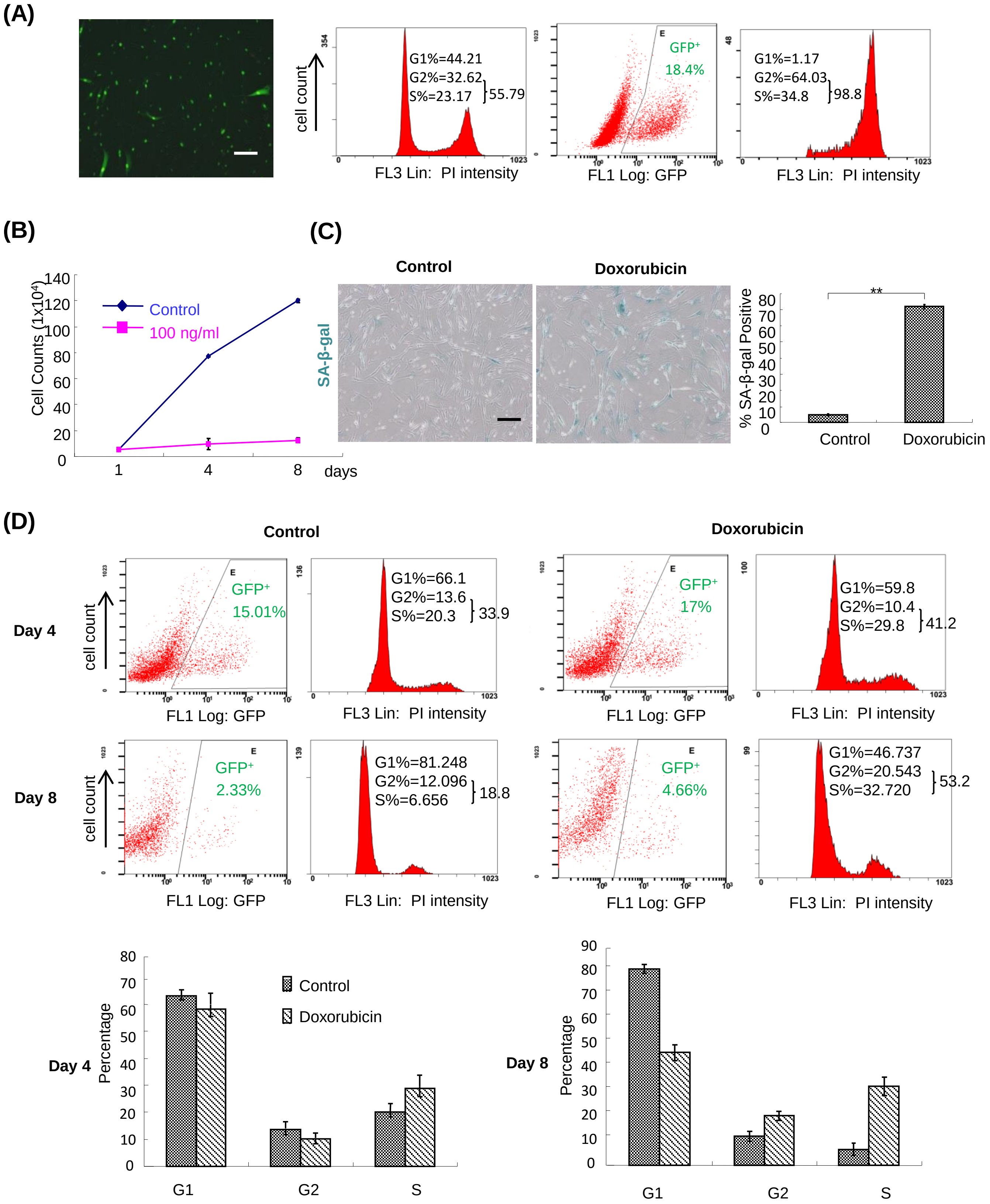

(A)
cell count
G1%=44.21
G2%=32.62
S%=23.17
55.79
FL3 Lin: PI intensity
GFP+
18.4%
FL1 Log: GFP
G1%=1.17
G2%=64.03
S%=34.8
98.8
FL3 Lin: PI intensity
(B)
Cell Counts (1x10^4)
140
120
100
80
60
40
20
0
1
4
8
days
Control
100 ng/ml
(C)
Control
Doxorubicin
SA-β-gal
% SA-β-gal Positive
80
70
60
50
40
30
20
10
0
**
Control
Doxorubicin
(D)
Control
Doxorubicin
Day 4
cell count
GFP+
15.01%
FL1 Log: GFP
G1%=66.1
G2%=13.6
S%=20.3
33.9
FL3 Lin: PI intensity
GFP+
17%
FL1 Log: GFP
G1%=59.8
G2%=10.4
S%=29.8
41.2
FL3 Lin: PI intensity
Day 8
cell count
GFP+
2.33%
FL1 Log: GFP
G1%=81.248
G2%=12.096
S%=6.656
18.8
FL3 Lin: PI intensity
GFP+
4.66%
FL1 Log: GFP
G1%=46.737
G2%=20.543
S%=32.720
53.2
FL3 Lin: PI intensity
Day 4
Percentage
80
70
60
50
40
30
20
10
0
Control
Doxorubicin
G1
G2
S
Day 8
Percentage
90
80
70
60
50
40
30
20
10
0
G1
G2
S
